# Relationship between authentic leadership and job satisfaction among undergraduate nursing professors*

**DOI:** 10.1590/1518-8345.7292.4476

**Published:** 2025-02-03

**Authors:** Cezar Augusto da Silva Flores, André Almeida de Moura, José Luis Guedes dos Santos, Alexandre Pazetto Balsanelli, Carmen Silvia Gabriel, Andrea Bernardes

**Affiliations:** 1 Universidade Federal de Mato Grosso, Instituto de Ciências da Saúde, Sinop, MT, Brazil; 2 Universidade Federal de Minas Gerais, Escola de Enfermagem, Belo Horizonte, MG, Brazil; 3 Universidade Federal de Santa Catarina, Departamento de Enfermagem, Florianópolis, SC, Brazil; 4 Universidade Federal de São Paulo, Departamento de Administração em Serviço de Saúde e Enfermagem, São Paulo, SP, Brazil; 5 Universidade de São Paulo, Escola de Enfermagem de Ribeirão Preto, PAHO/WHO Collaborating Centre for Nursing Research Development, Ribeirão Preto, SP, Brazil

**Keywords:** Leadership, Job Satisfaction, Nursing, Education, Education, Nursing, Faculty, Nursing

## Abstract

to analyze the relationship between the exercise of authentic leadership by coordinators and job satisfaction among professors of undergraduate nursing courses at federal public universities.

this is a quantitative study with an observational, analytical, and cross-sectional design. Twelve undergraduate nursing courses at public higher education institutions were chosen to participate in the study. The information collected was analyzed using descriptive statistical techniques, Spearman’s correlation test, and binary logistic regression analysis, with a 5% significance level.

the sample consisted of 179 professors who completed the Sociodemographic Questionnaire, the Authentic Leadership Questionnaire, and the Job Satisfaction Questionnaire. The professors rated the course coordinators as having high levels of authentic leadership practice. As for the binary logistic regression, only the Relational and Moral domain showed significance (p-value < 0.0001), indicating that when this domain is present, there is a 5.48-fold increase in the chance of professors being satisfied.

the results of the study indicate that the job satisfaction of professors in undergraduate nursing courses is influenced by the practice of authentic leadership on the part of the coordinators.

## Highlights

**(1)** Advances in the practice of authentic leadership in educational management.

**(2)** The instruments used are useful for assessing leadership and satisfaction.

**(3)** Relationships between authentic leadership and job satisfaction were identified.

**(4)** Measuring authentic leadership and job satisfaction in the educational environment.

## Introduction

In recent years, political, social, economic and cultural changes have transformed working relationships, highlighting the need to develop resilience, effectiveness and optimism in employees. In this context, training professionals in flexibility and improving their skills, especially in leadership, is essential in order to break away from outdated management models^(1-4)^.

Authentic leadership emerges as a theoretical model that emphasizes the moral and ethical character of the leader, promoting transparency in working relationships and generating respect and trust through behaviors aligned with personal values and convictions. This approach focuses on establishing positive relationships between leaders and those they lead, based on the leader’s loyalty to their needs, desires, beliefs and emotions^(2,5-6)^.

The theoretical model of authentic leadership is characterized by transparent communication, self-awareness, and balance in decision-making, prioritizing collective interests and the development of employees, with the potential to positively influence the work environment, especially the well-being, performance and psychological factor of followers^(2,5-6)^.

Job satisfaction, on the other hand, is the professional’s perception of the relationship between their expectations and what they get in the workplace, resulting in emotional pleasure. There is a consensus that job satisfaction influences overall satisfaction, as emotions and attitudes at work affect personal life^(7-8)^.

In the educational context, research on job satisfaction is scarce and often incorporates theories and concepts from other areas, disregarding the particularities of this environment^(9)^. Despite the scarcity of research on job satisfaction in the educational context, existing studies indicate that social relationships in the workplace are more influential on satisfaction than salary rewards^(2,9-10)^. The relationship between authentic leadership and job satisfaction is well documented in various fields, but specific evidence on nursing professors is lacking^(2)^.

In view of the above, this study is justified in exploring in detail the relationship between the application of the concept of authentic leadership by undergraduate course coordinators and its impact on the job satisfaction of nursing professors. It is important to emphasize that no studies have been identified that address this issue, making this a pioneering study.

Based on the hypothesis that the authentic leadership exercised by the coordinators of undergraduate nursing courses is associated with the job satisfaction of professors, this research has the potential to reveal information not yet explored by the scientific community, with the caveat that its results can guide educational institutions in promoting professional growth, based on high academic standards.

Thus, the aim of this study was to analyse the relationship between the exercise of authentic leadership by coordinators and job satisfaction among undergraduate nursing professors at federal public universities.

## Method

### Type of study

This is a quantitative study with an observational, analytical and cross-sectional design. In this way, the Strengthening the Reporting of Observational Studies in Epidemiology (STROBE) recommendations guide was used as a tool to support the communication of the results shown in this study^(11)^.

### Study setting

The research took place in the undergraduate nursing programs of federal higher education institutions located in the Midwest region of Brazil, covering the states of Goiás, Mato Grosso and Mato Grosso do Sul and the Federal District (Brasília).

This region was selected due to the scarcity of scientific research in the field of academic management and satisfaction in the workplace, especially in the academic context. In addition, this region covers a wide area in Brazil, with few research groups dedicated to producing knowledge in the area of management and leadership in nursing.

### Study population

The study population was defined based on a sample made up of professors working on undergraduate nursing courses at these universities, considering the following inclusion criteria: being a professor with permanent or interim institutional ties to these universities and having a coordinator with at least six months’ experience in the academic management of the course.

The sample size was calculated for a finite population. Given that the eligible population for the study consisted of 354 professors, the sample size was estimated at 184 participants, with a margin of error of 5% and a confidence level of 95%. However, the survey covered 179 professors from 12 nursing courses offered by seven higher education institutions who answered the questionnaires. As a result of not fully meeting the sample size, the margin of error was recalculated for the number of responses collected (179), which was 5.1%, with a 95% confidence level. It is confirmed that the reduction in the sample did not cause any loss to the results of the survey.

### Data collection and period

Data collection began in August and ended in November 2021 using Google Forms^®^, which was made available via electronic links or messaging applications. Participants were given seven days to respond to the questionnaires after the invitation was sent. In the event of a non-response, reminders were sent seven days apart until the fifth attempt.

Participants received invitations by email or message with links to a private platform, where they had to fill in the Informed Consent Form (ICF) before accessing the survey questionnaires.

### Data collection instruments

Firstly, the researcher prepared a sociodemographic questionnaire to obtain information on the characterization of the research subjects, which helped to contextualize the results.

To assess the authenticity of the leaders, the study participants were asked to fill in the Authentic Leadership Questionnaire (ALQ)^(12)^, validated by researchers in Brazil^(13)^. The ALQ has two versions: the first, entitled “Evaluating my Way of Leading” (SELF), in which leaders carry out a self-assessment of their level of authentic leadership; and the second, entitled “Evaluating your Leader” (RATER), in which those being led assess the degree of authentic leadership of their leaders^(12-13)^, this being the version used in this research.

After the instrument had been validated for use in Brazilian Portuguese, the two versions (SELF and RATER) were adapted, with the RATER version being reduced from 16 to 13 items distributed in three domains: Relational and Moral, Balanced Information Processing and Self-awareness^(13)^.

Relational and Moral are understood to be when the leader strives to establish transparent and sincere relationships, based on solid moral and ethical values, with a view to the well-being and growth of those they lead. Balanced Information Processing involves the objective analysis of all relevant information before the leader makes a decision. This approach seeks to consider various aspects and points of view, ensuring informed and impartial decision-making. Self-Awareness refers to the leader’s ability to recognize himself as a human being, having discernment about his abilities and limitations^(12-13)^.

Each item is evaluated on an ordinal frequency scale, ranging from zero to four. For each item, the participant chooses one of five options on a Likert scale, whose values from 0 to 4 represent the degree of authenticity of the leader: 0 = rarely/never; 1 = once in a while; 2 = sometimes; 3 = often; 4 = often/almost always. Thus, higher scores indicate a greater perception of authenticity, both on the part of the leader and the person being led^(12-13)^.

To collect information on job satisfaction, we used the Job Satisfaction Questionnaire (S20/23) adapted for Brazilian Portuguese^(14)^. The questionnaire consists of 20 items divided into three categories/factors and was evaluated using a five-point Likert scale, where 1 indicates totally dissatisfied and 5 indicates totally satisfied. In order to assess overall satisfaction, the median was calculated for each of the factors, considering that factors with a median greater than three points indicated a high tendency towards satisfaction^(14)^.

### Data processing and analysis

The IBM Statistical Package for the Social Sciences (SPSS) software, version 22 in Portuguese, was used to process the data collected. In the statistical analysis, descriptive and inferential analyses were carried out to assess the relationships between the variables.

Initially, a descriptive survey was carried out to assess professors’ perceptions of authentic leadership, using the RATER version of the questionnaire and considering its domains (Relational and Moral, Balanced Information Processing and Self-Awareness), as well as total authentic leadership. The data was subjected to the Kolmogorov-Smirnov normality test with Lilliefors correction and, when it was found that it did not follow a normal distribution, the results were expressed in terms of position, using scores and medians.

Spearman’s correlation test (*rho*) was used to investigate the relationship between authentic leadership (ALQ) and job satisfaction, along with its associated domains and factors. This is a non-parametric statistical technique suitable for assessing the relationship between ordinal variables or when the data does not have a normal distribution^(15)^.

When examining the relationships between the components of job satisfaction and the different aspects of authentic leadership, it was decided to conduct binomial logistic regression analysis.

Binomial logistic regression analysis makes it possible to estimate the probability of a specific categorical outcome (job satisfaction) based on one or more predictive factors (authentic leadership), which can be categorical or continuous^(16)^. Before applying the model, the multicollinearity between the variables was assessed using the Variance Inflation Factor (VIF)^(16)^.

To this end, all the variables were discretized into dichotomous categories in order to quantify the odds ratios associated with job satisfaction in relation to each domain of authentic leadership. This conversion was made from multinominal to binomial data, since the original data showed considerable dispersion. In this way, the data was grouped into two distinct categories: “no or absent”, represented by the value zero (0), and “yes or present”, which received the value one (1). This procedure made the analysis more solid and robust.

The quality of the fit was checked by the Hosmer and Lemeshow test, which measures the degree of accuracy of the logistic model and, finally, the Nagelkerke R2 (adjusted), which indicates how much of the variation in the result can be explained by the predictor variable (authentic leadership). The higher the R2, the better the fit of the model^(16)^.

### Ethical aspects

Observing the ethical principles governing research with human participants, the project was submitted for analysis by the Research Ethics Committee of the Ribeirão Preto School of Nursing, part of the University of São Paulo (USP). This was done in order to meet the requirements set out in Resolution 466/2012 of the National Health Council, which establishes the guidelines for conducting studies involving human beings. Consequently, the project received approval, according to opinion number 3.506.984/2019.

The lead researcher was in charge of data collection, ensuring that the anonymity of the participants was preserved and that all information was treated confidentially. In addition, the results were presented in aggregate form, without ever individualizing or identifying the participants.

## Results

The research was carried out with 12 nursing courses offered at seven higher education institutions. 354 professors were eligible to take part in the study and of these, 179 answered the questionnaires (50.560%). Professionals with less than six months’ employment were excluded from the sample. Of the other professors invited to take part in the study, three said, in response to the e-mail invitation, that they felt uncomfortable evaluating their coordinator, which led them to decline the invitation to take part in the study. There was no response from the other respondents.

The majority of respondents were female (86%), taught subjects in the Nursing Sciences area (83.2%), had completed postgraduate studies (94.4%) and were civil servants (89.9%), as shown in Table 1. It is important to note that the answers to the sociodemographic data on the form provided were not mandatory. As a result, some items were not filled in by all the research participants, resulting in items with fewer than 179 respondents.

**Table 1 -:** Presentation of sociodemographic data of undergraduate nursing professors (N = 179). Midwest, Brazil, 2021

	**Categories**	**Type N (%)**
Age	20 to 39 years	96 (53.630)
	40 to 69 years	79 (44.130)
	Total	175 (97.760)
Sex	Male	25 (13.970)
	Female	154 (86.030)
	Total	179 (100)
Subjects	Biological and Health Sciences and Human and Social Sciences	29 (16.200)
	Nursing Sciences	149 (83.240)
	Total	178 (99.440)
Level of training	Bachelor’s degree and/or specialization	10 (5.590)
	Master’s degree and/or doctorate	169 (94.410)
	Total	179 (100)
Type of employment	Contracted	161 (89.940)
	Contracted and/or volunteer	18 (10.060)
	Total	179 (100)
Working hours	20, 30 or 40 hours	27 (15.090)
	40 hours with exclusive dedication	152 (84.910)
	Total	179 (100)
Years of teaching in the nursing course	01 to 09 years	73 (40.780)
	10 to 19 years	77 (43.020)
	20 to 39 years	29 (16.200)
	Total	179 (100)
Wage income*	1 to 9 minimum wages^†^	88 (49.160)
	9.1 to 12 minimum wages^‡^	47 (26.260)
	12.1 to 15 minimum wages or more^§^	44 (24.580)
	Total	179 (100)
Have you ever held a management position	No	93 (51.960)
	Yes	86 (48.040)
	Total	179 (100)

*Minimum wage R$ 1.100.00, Brazil, 2021; ^†^Salary between R$ 1.100.00 and R$ 9.900.00; ^‡^Salary between R$ 10.010.00 and R$ 13.200.00; ^§^Salary between R$ 13.310.00 and R$ 16.500.00 or more

The professors’ understanding of the coordinators’ authentic leadership was assessed (Table 2). In order to facilitate the analysis of the scores, the participants were categorized into three levels (low, medium and high) based on the total value of the score, considering that there were a large number of subjects (179). It is important to note that the questionnaire has a difference in the number of questions between the domains. Thus, the RATER version could reach a maximum score of 52 points. The analysis revealed that the majority of professors (72.000%) considered their coordinators to have a high level of authentic leadership in the academic administration of the course, with a median score of 42 points. Only 3.300% of professors rated their coordinators as having low authentic leadership.

**Table 2 -:** Professors’ perception of coordinators’ authentic leadership (N = 179). Midwest, Brazil, 2021

	**Score**	**n**	**%**	**Median**
Relational and Moral	0 – 9*	6	3.400	23
	10 – 18^†^	40	22.300	
	19 – 28^‡^	133	74.300	
Balanced Information Processing	0 – 4*	13	7.300	10
	5 – 8^†^	44	24.500	
	9 – 12^‡^	122	68.200	
Self-awareness	0 – 4*	11	6.200	9
	5 – 8^†^	56	31.200	
	9 – 12^‡^	112	62.600	
Total Authentic Leadership	0 – 17*	6	3.300	42
	18 – 34^†^	44	24.700	
	35 – 52^‡^	129	72.000	

*Low score for authentic leadership; ^†^Medium score for authentic leadership; ^‡^High score for authentic leadership

When analyzing the correlation between the factors of job satisfaction and the areas or dimensions of authentic leadership, in the Relational and Moral domain there was a positive correlation with all the elements of job satisfaction, with the highest correlation found being between Relational and Moral and satisfaction with hierarchical relations (SRH) (*rho* = 0.416, p < 0.0001). Balanced Information Processing showed a significant correlation only with SRH (*rho* = 0.335, p < 0.0001) and with total job satisfaction (*rho* = 0.235, p = 0.002). In the Self-Consciousness domain, there was a significant positive correlation between SRH (*rho* = 0.366, p < 0.0001), intrinsic job satisfaction (SIT) (*rho* = 0.215, p = 0.004) and total job satisfaction (*rho* = 0.292, p < 0.0001). Finally, when investigating total authentic leadership, there was a significant correlation with all the job satisfaction indicators, the highest being with the SRH factor (*rho* = 0.402, p < 0.0001) (Table 3).

**Table 3 -:** Correlation between job satisfaction and authentic leadership (N = 179). Midwest, Brazil, 2021

**Variables**	**Job satisfaction**							
	SRH*		**SAFT** ^†^		**SIT** ^‡^		**TOTAL**	
	***rho***	**p-value**	***rho***	**p-value**	***rho***	**p-value**	***rho***	**p-value**
Relational and Moral	0.416^§^	<0.0001	0.210^||^	0.005	0.257^§^	0.001	0.365^§^	<0.0001
Balanced Information Processing	0.335^§^	<0.0001	0.126	0.093	0.110	0.142	0.235^||^	0.002
Self-awareness	0.366^§^	<0.0001	0.127	0.089	0.215^||^	0.004	0.292^§^	<0.0001
Total Authentic Leadership	0.402^§^	<0.0001	0.176^||^	0.018	0.226^||^	0.002	0.331^§^	<0.0001

*SRH = Satisfaction with hierarchical relations; ^†^SAFT = Satisfaction with physical work environment; ^‡^SIT = Intrinsic job satisfaction; ^§^Spearman correlation significant at 1% level; ^||^Spearman correlation significant at 5% level

Based on the correlations found between authentic leadership and job satisfaction, it can be inferred that, according to the professors’ perception, the adoption of authentic leadership practices in the administration of undergraduate courses is related to improved satisfaction with teaching work.

Binary logistic regression was used to analyze the relationship between the dependent variable Job satisfaction and the independent variables (domains of authentic leadership). The model proved to be well adjusted, with a statistically significant quality of fit parameter [Hosmer and Lemeshow test: ^X2^(2) = 0.124, p = 0.940,^R2^ Nagelkerke = 0.130 and VIF=1.140]. The model produced Beta coefficients (β) that indicate the influence of each domain of authentic leadership on job satisfaction: Relational and Moral (β = 1.700), Balanced Information Processing (β = 0.270), Self-awareness (β = 0.060) and total authentic leadership (β = 0.480) (as illustrated in Table 4).

**Table 4 -:** Odds ratios between authentic leadership and job satisfaction (N = 179). Midwest, Brazil, 2021

**Variables**		**Job satisfaction**		B*	**Odds Ratio (95%)**	**p-value**
		**Dissatisfied**	**Satisfied**			
		**N(%)**	**N(%)**			
Relational and Moral	No	21(33.900)	10(8.500)	1.700	Reference	<0.0001^†^
	Yes	41(66.100)	107(91.500)		5.480 (2.380 - 12.630)	
Balanced information processing	No	22(35.500)	20(17.100)	0.270	Reference	0.961
	Yes	40(64.500)	97(82.900)		1.030 (0.340 - 3.070)	
Self-awareness	No	27(43.500)	24(20.500)	0.060	Reference	0.926
	Yes	35(56.500)	93(79.500)		1.060 (0.310 - 3.570)	
Total authentic leadership	No	24(38.700)	15(12.800)	0.480	Reference	0.521
	Yes	38(61.300)	102(87.200)		1.610 (0.370 - 7.070)	

*Beta coefficient; ^†^Significant at p ≤ 0.001

Running the binary logistic regression model showed that only the Relational and moral domain was statistically significant (p-value < 0.0001), indicating that when the Relational and moral domain is present, there is a 5.480-fold increase in the chance of professors being satisfied with their work. When analyzing the distribution of Professors in the Relational and moral domain with job satisfaction, the high rates of this variable are among the satisfaction group (n=107; 91.500%).

## Discussion

Based on the sociodemographic data of the professors taking part in the survey, it was found that the majority are aged between 20 and 39, female, teach subjects in the area of nursing sciences, have a master’s degree and/or doctorate, are civil servants, work 40-hour shifts with ED, have been working in higher education for between 10 and 19 years, have a salary of between 1 and 12 minimum wages and have never held management positions.

Based on the analysis of the relationship between the exercise of authentic leadership by coordinators and job satisfaction among professors of undergraduate nursing courses at federal public universities, it was found that the professors involved in the research gave the highest ratings on the scale (“often, almost always”) to all the domains of authentic leadership, as well as to the overall assessment of authentic leadership. These results suggest that the participants had a highly positive perception of authentic leadership. This conclusion is supported by previous research that has also highlighted the prevalence of leaders with substantial levels of authenticity in various domains of authentic leadership^(17-20)^.

In addition, it is important to note that those who perceive authenticity in their leaders tend to have greater hope, optimism, confidence and resilience^(18)^.

Authentic leadership in education is seen as highly positive in this research; the results indicate that the Professors rated the authenticity measures high in all domains, suggesting that the coordinators have the attitudes of a genuinely authentic leader. This is relevant to the educational context, as it indicates that this model of leadership represents value in this environment and leaders who use authentic leadership can have followers who are more engaged in implementing the necessary changes.

Authentic leadership plays a fundamental role in the educational context by encouraging positive behaviour from professors and fostering a work environment characterized by trust and optimism. Authentic leaders have the ability to influence the educational environment, increasing professors’ effectiveness and helping them deal with different situations, which reinforces commitment and reduces dissatisfaction^(19)^.

The practice of authentic leadership establishes a favorable working environment that encourages the autonomy of those being led and boosts motivation, especially when the leader’s decisions are fair and appropriate for the institution^(20)^. Making decisions based on ethical principles is a fundamental component of the actions of leaders in the educational context, involving principles of justice, equality and democracy. Therefore, promoting authentic leadership can be an important strategy for improving the educational environment, professor satisfaction and, consequently, the quality of teaching^(2)^.

When evaluating the relationship between authentic leadership and professor job satisfaction, it can be seen that the presence of authentic leadership on the part of coordinators results in greater satisfaction on the part of professors. This relationship is also seen in other areas, such as nursing, where nurses rate their managers highly in terms of authentic leadership, resulting in less emotional exhaustion and greater job satisfaction^(21)^.

In the field of education, there is a positive correlation between the authenticity of the principal and the trust and engagement of professors^(22)^. Another study found that authentic leadership and job satisfaction among civil servants are positively correlated with motivation, and that these factors have different effects on the engagement of these professionals^(23)^. In addition, one study indicated a moderate and significant negative correlation between authentic leadership and professors’ work pressure, i.e. the more authentic the leader, the lower the level of stress perceived by those they lead^(24)^.

Therefore, by adopting the theoretical model of authentic leadership, it is possible to establish a transparent relationship with professors, which can improve motivation, satisfaction and mutual trust. This creates an environment of safety and respect, strengthening the ethics and morals of professionals, resulting in professors’ greater perception of organizational support^(25)^.

Based on the data presented, contemporary leadership models are related to job satisfaction, albeit relatively. The results indicate that authentic leadership has a direct impact on nurse professors’ job satisfaction from different perspectives. However, leaders need to be aware that the organization will not always be able to meet all the perspectives of those they lead, and need to strive to offer a reliable, flexible working atmosphere and promote positive working relationships.

It was observed that authentic leadership explains only 13.500% of the total variation in job satisfaction, which is considered representative for a relatively new theoretical model such as authentic leadership. After analysis, it is possible to state that there is a significant association between authentic leadership and job satisfaction, especially in the Relational and Moral domain, since it increases the chance of feeling satisfied in professional practice by 5.48 times. Although the other domains of authentic leadership were not statistically significant, they still show a positive relationship with job satisfaction among nursing professors at federal universities in the Brazilian Midwest.

Other recent studies have also highlighted the same domains of authentic leadership, which confirms the importance of these factors for the construct^(22,24,26-28)^. However, some studies have differed in their assessment of the domains of authentic leadership. For example, a study of recently graduated nurses found that leader self-awareness was weakly correlated with work engagement^(29)^. In another study carried out in schools in Pakistan, there was variation in the professors’ assessment of the domains, with the majority rating the principal as Relational and Moral, but in some schools, the principal was assessed as having Balanced Information Processing or Self-awareness^(28)^. However, a study with academic leaders showed consistency in all dimensions of authentic leadership, except in the Relational and Moral domain^(27)^.

Although the literature on democratic education does not use the same terms as authentic leadership, such as Relational and Moral, Balanced Information Processing and Self-Awareness, the concepts underlying these domains, such as the culture of honesty, commitment and participatory judgment making, are essential components in the democratic educational environment^(27)^.

Based on the results presented, it can be concluded that the theoretical model of Authentic Leadership is highly desirable and effective for the development of the human factor, leading to positive results in organizations, including educational institutions. According to the professors’ perception, the three domains of Authentic Leadership were considered important for nursing courses at federal universities. Therefore, the adoption of this theoretical model is seen as important for the educational context.

This study had some limitations, such as the exclusive use of online research and convenience sampling. Due to the heterogeneity of the sample, the results cannot be generalized to professors working in federal public universities in other regions of Brazil. Therefore, it is necessary to carry out national surveys with larger samples to validate the results found.

## Conclusion

The results of the study indicated a significant positive association, with the job satisfaction of professors in undergraduate nursing courses being influenced by the practice of authentic leadership on the part of the coordinators, with emphasis on the three variables: Relational and Moral, Balanced Information Processing and Self-Awareness. This confirms the hypothesis raised in the research and suggests that authentic leadership behaviors can positively influence the teaching work environment. This understanding can lead to improved management, through more assertive decisions in the educational environment.

The study highlights the importance of authentic leadership as a significant factor in the job satisfaction of undergraduate nursing Professors at federal public universities in the Brazilian Midwest. Although the association is modest, authentic leadership, even though it is a relatively recent theoretical model, is significantly representative.

The Relational and Moral domains showed a significant relationship with professors’ job satisfaction, which reinforces the importance of these factors for the construct. However, it is important to remember that there are other factors which influence job satisfaction and which need to be investigated in future studies. Authentic leadership lays a solid foundation for the adoption of contemporary and innovative leadership models in the educational environment, and ongoing guidance and integration are fundamental to ensuring a positive and satisfying working environment.

The results obtained confirm the relevance of authentic leadership in education, especially in the context of nursing education, and open up new opportunities for future research into management and leadership models in the educational field. In addition, these findings provide valuable guidance for coordinators, managers and educational leaders, enabling them to base their management and leadership practices on the principles of authentic leadership. This, in turn, contributes to the promotion of more positive working relationships and increased levels of satisfaction in the workplace.
