# Supplementary material for: Relationship between authentic leadership and job satisfaction among undergraduate nursing professors
Source: Rev Lat Am Enfermagem. 2025 Feb 3;33:e4476. doi: 10.1590/1518-8345.7292.4476 (PMC11789744; doi:10.1590/1518-8345.7292.4476)
Supplement: Translation

# Translation: Relación entre el liderazgo auténtico y la satisfacción en el trabajo de profesores de pregrado en enfermería*

**DOI:** 10.1590/1518-8345.7292.4475

**Authors:** Cezar Augusto da Silva Flores, André Almeida de Moura, José Luis Guedes dos Santos, Alexandre Pazetto Balsanelli, Carmen Silvia Gabriel, Andrea Bernardes

**Affiliations:** 1 Universidade Federal de Mato Grosso, Instituto de Ciências da Saúde, Sinop, MT, Brasil; 2 Universidade Federal de Minas Gerais, Escola de Enfermagem, Belo Horizonte, MG, Brasil; 3 Universidade Federal de Santa Catarina, Departamento de Enfermagem, Florianópolis, SC, Brasil; 4 Universidade Federal de São Paulo, Departamento de Administração em Serviço de Saúde e Enfermagem, São Paulo, SP, Brasil; 5 Universidade de São Paulo, Escola de Enfermagem de Ribeirão Preto, PAHO/WHO Collaborating Centre for Nursing Research Development, Ribeirão Preto, SP, Brasil

**Keywords:** Liderazgo, Satisfacción en el Trabajo, Enfermería, Educación, Educación em Enfermería, Docentes de Enfermería

## Abstract

analizar la relación entre el ejercicio del liderazgo auténtico por parte de los coordinadores y la satisfacción en el trabajo de los profesores de los cursos de pregrado en enfermería de universidades públicas federales.

se trata de un estudio con enfoque cuantitativo, diseño observacional analítico y transversal. Se seleccionaron doce cursos de pregrado en enfermería en Instituciones de Educación Superior públicas para participar en el estudio. La información recopilada fue analizada utilizando técnicas de estadística descriptiva, prueba de correlación de Spearman y análisis de regresión logística binaria, con un nivel de significancia del 5%.

la muestra consistió en 179 profesores que completaron el Cuestionario Sociodemográfico, el Cuestionario de Liderazgo Auténtico y el Cuestionario de Satisfacción en el Trabajo. Los profesores evaluaron que los coordinadores de los cursos presentaban altos niveles de práctica de liderazgo auténtico. En cuanto a la regresión logística binaria, se observó que solo el dominio Relacional y Moral presentó significancia (valor p < 0,0001), indicando que cuando este dominio está presente, la probabilidad de que los profesores estén satisfechos aumenta 5,48 veces.

los resultados del estudio indicaron que la satisfacción en el trabajo de los profesores en los cursos de pregrado en enfermería está influenciada por la práctica del liderazgo auténtico por parte de los coordinadores.

## Destacados

**(1)** Avances en la práctica del liderazgo auténtico en la gestión educativa.

**(2)** Los instrumentos utilizados son útiles para evaluar el liderazgo y la satisfacción.

**(3)** Se identificaron relaciones entre el liderazgo auténtico y la satisfacción en el trabajo.

**(4)** Medir el liderazgo auténtico y la satisfacción en el trabajo en el entorno educativo.

## Introducción

En los últimos años, los cambios políticos, sociales, económicos y culturales han transformado las relaciones laborales, destacando la necesidad de desarrollar resiliencia, eficacia y optimismo en los empleados. En este contexto, capacitar a los profesionales con flexibilidad y mejora de habilidades, especialmente en liderazgo, se vuelve esencial para romper con modelos de gestión obsoletos^(1-4)^.

El liderazgo auténtico emerge como un modelo teórico que enfatiza el carácter moral y ético del líder, promoviendo la transparencia en las relaciones laborales y generando respeto y confianza mediante comportamientos alineados con los valores y convicciones personales. Este enfoque se centra en establecer relaciones positivas entre líderes y seguidores, basadas en la fidelidad del líder a sus necesidades, deseos, creencias y emociones^(2,5-6)^.

El modelo teórico del liderazgo auténtico se caracteriza por la comunicación transparente, la autoconciencia y el equilibrio en la toma de decisiones, priorizando los intereses colectivos y el desarrollo de los colaboradores, con el potencial de influir positivamente en el entorno laboral, especialmente en el bienestar, el desempeño y el factor psicológico de los seguidores^(2,5-6)^.

Por su parte, la satisfacción en el trabajo es la percepción del profesional sobre la relación entre sus expectativas y lo que obtiene en el entorno laboral, resultando en placer emocional. Existe consenso en que la satisfacción en el trabajo influye en la satisfacción general, ya que las emociones y actitudes en el trabajo afectan la vida personal^(7-8)^.

En el contexto educativo, las investigaciones sobre satisfacción laboral son escasas y, a menudo, incorporan teorías y conceptos de otras áreas, sin considerar las particularidades de este entorno^(9)^. A pesar de la escasez de estudios sobre la satisfacción laboral en el contexto educativo, las investigaciones existentes indican que las relaciones sociales en el entorno laboral influyen más en la satisfacción que las recompensas salariales^(2,9-10)^. La relación entre el liderazgo auténtico y la satisfacción en el trabajo está bien documentada en diversas áreas, pero faltan evidencias específicas sobre los profesores de enfermería^(2)^.

Dado lo expuesto, este estudio se justifica al explorar minuciosamente la relación entre la aplicación del concepto de liderazgo auténtico por parte de los coordinadores de los cursos de pregrado y su impacto en la satisfacción laboral de los profesores de los cursos de enfermería. Es importante señalar que no se han identificado estudios que aborden esta temática, lo que hace que este estudio sea precursor.

Partiendo de la hipótesis de que el liderazgo auténtico ejercido por los coordinadores de los cursos de pregrado en enfermería presenta una asociación con la satisfacción en el trabajo de los docentes, esta investigación tiene el potencial de revelar información aún no explorada por la comunidad científica, con la salvedad de que sus resultados pueden guiar a las instituciones educativas en la promoción del crecimiento profesional, basándose en estándares académicos elevados.

De esta manera, el presente estudio tuvo como objetivo analizar la relación entre el ejercicio del liderazgo auténtico por parte de los coordinadores y la satisfacción en el trabajo de los profesores de los cursos de pregrado en enfermería de universidades públicas federales.

## Método

### Tipo do estudio

Se trata de un estudio con enfoque cuantitativo, diseño observacional analítico y transversal. De este modo, en la presente investigación se utilizó la guía de recomendaciones *Strengthening the Reporting of Observational Studies in Epidemiology* (STROBE) como herramienta para respaldar la comunicación de los resultados evidenciados^(11)^.

### Lugar del estudio

La investigación se llevó a cabo en los programas de pregrado en enfermería de instituciones federales de educación superior ubicadas en la región *Centro-Oeste* de Brasil, abarcando los estados de Goiás, Mato Grosso y Mato Grosso do Sul, y el Distrito Federal (Brasilia).

Esta región fue seleccionada debido a la escasez de investigaciones científicas en el campo de la gestión académica y la satisfacción en el entorno laboral, especialmente en el contexto académico. Además, esta región cubre una amplia extensión territorial en Brasil, con pocos grupos de investigación dedicados a la producción de conocimiento en el área de la gestión y liderazgo en enfermería.

### Población del estudio

La población del estudio se definió a partir de una muestra, compuesta por los profesores que trabajan o trabajaban en los cursos de pregrado en enfermería de estas universidades, considerando como criterio de inclusión: ser docente con vínculo institucional efectivo o interino con dichas universidades y contar con un coordinador que posea, como mínimo, seis meses de experiencia en la gestión académica del curso.

Se realizó el cálculo del tamaño de la muestra para una población finita. Dado que la población elegible para el estudio consistía en 354 profesores, se estimó el tamaño de la muestra en 184 participantes, con un margen de error del 5% y un nivel de confianza del 95%. Sin embargo, la investigación abarcó a 179 profesores de 12 cursos de Enfermería ofrecidos por siete Instituciones de Educación Superior que respondieron a los cuestionarios. Debido a que no se alcanzó el tamaño total de la muestra, se recalculó el margen de error para el número de respuestas recolectadas (179), siendo este de 5,1%, con un nivel de confianza del 95%. Se confirma que la disminución de la muestra no causó pérdidas en el resultado de la investigación.

### Recolección de datos y período

La recolección de datos comenzó en el mes de agosto y finalizó en noviembre de 2021, y se llevó a cabo mediante *Google Forms*®, que se puso a disposición por medio de enlaces electrónicos o aplicaciones de mensajería. Los participantes recibieron un plazo de siete días para responder a los cuestionarios después de recibir la invitación. En caso de no respuesta, se enviaron recordatorios con un intervalo de siete días hasta el quinto intento.

Los participantes recibieron invitaciones por correo electrónico o mensaje con enlaces a una plataforma privada, donde debían firmar el Consentimiento Informado (CI) antes de acceder a los cuestionarios de la investigación.

### Instrumentos para la recolección de datos

En primer lugar, el investigador elaboró un cuestionario sociodemográfico para obtener información sobre la caracterización de los sujetos de la investigación, lo que contribuyó a contextualizar los resultados.

Para evaluar la autenticidad de los líderes, los participantes del estudio fueron invitados a completar el Cuestionario de Liderazgo Auténtico (ALQ)^(12)^, validado por investigadores en Brasil^(13)^. El ALQ tiene dos versiones: la primera, titulada “Evaluando mi Modo de Liderar” (*SELF*), en la que los líderes realizan una autoevaluación de su nivel de liderazgo auténtico; y la segunda, denominada “Evaluando a su Líder” (*RATER*), en la que los subordinados evalúan el grado de liderazgo auténtico de sus líderes^(12-13)^, siendo esta la versión utilizada en la presente investigación.

Una vez que el instrumento fue validado para su uso en portugués de Brasil, se realizó la adaptación de ambas versiones (*SELF* y *RATER*), reduciendo la versión *RATER* de 16 a 13 ítems distribuidos en tres dominios: Relacional y Moral, Procesamiento Equilibrado de Información y Autoconciencia^(13)^.

Se entiende por Relacional y Moral cuando el líder se esfuerza por establecer relaciones transparentes y sinceras, basadas en sólidos valores morales y éticos, con el objetivo de fomentar el bienestar y el crecimiento de sus subordinados. El Procesamiento Equilibrado de Información implica el análisis objetivo de toda la información relevante antes de que el líder tome una decisión. Este enfoque busca considerar diversos aspectos y puntos de vista, garantizando una toma de decisiones fundamentada e imparcial. La Autoconciencia se refiere a la capacidad del líder para reconocerse como ser humano, con discernimiento sobre sus habilidades y limitaciones^(12-13)^.

Cada ítem se evalúa en una escala ordinal de frecuencia, que varía de cero a cuatro. Para cada ítem, el participante elige una de las cinco opciones en una escala Likert, cuyos valores de 0 a 4 representan el grado de autenticidad del líder, siendo: 0 = raramente/nunca; 1 = de vez en cuando; 2 = a veces; 3 = frecuentemente; 4 = muchas veces/casi siempre. De esta manera, puntuaciones más altas indican una mayor percepción de autenticidad, tanto por parte del líder como del subordinado^(12-13)^.

Para recopilar información sobre la satisfacción en el trabajo, se utilizó el Cuestionario de Satisfacción en el Trabajo (S20/23) adaptado al portugués de Brasil^(14)^. El cuestionario consiste en 20 ítems divididos en tres categorías/factores y fue evaluado utilizando una escala Likert de cinco puntos, donde 1 indica totalmente insatisfecho y 5 indica totalmente satisfecho. Para evaluar la satisfacción global, se calculó la mediana para cada uno de los factores, considerando que los factores con una mediana superior a tres puntos indicaban una alta tendencia a la satisfacción^(14)^.

### Tratamiento y análisis de los datos

Se utilizó el *software* IBM *Statistical Package for the Social Sciences* (SPSS), versión 22 en portugués, para el tratamiento de los datos recolectados. En el análisis estadístico se realizaron análisis descriptivos e inferenciales para evaluar las relaciones entre las variables.

Inicialmente, se llevó a cabo un análisis descriptivo para evaluar la percepción de los profesores sobre el liderazgo auténtico, utilizando la versión *RATER* del cuestionario y considerando sus dominios (Relacional y Moral, Procesamiento Equilibrado de Información y Autoconciencia), así como el liderazgo auténtico total. Los datos se sometieron a la prueba de normalidad de Kolmogorov-Smirnov con la corrección de Lilliefors y, al observar que no seguían una distribución normal, los resultados se expresaron en términos de posición, utilizando puntuaciones y medianas.

La prueba de correlación de Spearman (*rho*) se utilizó para investigar la relación entre el liderazgo auténtico (ALQ) y la satisfacción en el trabajo, junto con sus dominios y factores asociados. Esta es una técnica estadística no paramétrica adecuada para evaluar la relación entre variables ordinales o cuando los datos no tienen distribución normal^(15)^.

Al examinar las relaciones entre los componentes de la satisfacción en el trabajo y los diferentes aspectos del liderazgo auténtico, se optó por realizar el análisis de regresión logística binomial.

El análisis de regresión logística binomial permite estimar la probabilidad de un resultado categórico específico (satisfacción en el trabajo) en función de uno o más factores predictivos (liderazgo auténtico), que pueden ser categóricos o continuos^(16)^. Antes de aplicar el modelo, se evaluó la multicolinealidad entre las variables mediante el Factor de Inflación de Varianza (VIF)^(16)^.

Para este propósito, todas las variables se discretizaron en categorías dicotómicas, con el fin de cuantificar las razones de probabilidades asociadas a la satisfacción en el trabajo en relación con cada dominio del liderazgo auténtico. Se realizó esta conversión de datos multinomiales a binomiales, dado que los datos originales presentaban una dispersión considerable. De esta manera, los datos se agruparon en dos categorías distintas: “no o ausente”, representada por el valor cero (0), y “sí o presente”, que recibió el valor uno (1). Este procedimiento hizo que el análisis fuera más sólido y robusto.

La calidad del ajuste se verificó mediante la prueba de Hosmer y Lemeshow, que mide el grado de precisión del modelo logístico, y finalmente, el R2 de Nagelkerke (ajustado), que indica cuánto de la variación en el resultado puede explicarse por la variable predictora (liderazgo auténtico). Cuanto mayor es el R2, mejor es el ajuste del modelo^(16)^.

### Aspectos éticos

Observando los principios éticos que rigen la investigación con participantes humanos, el proyecto fue enviado para su análisis al Comité de Ética en Investigación de la *Escola de Enfermagem de Ribeirão Preto*, perteneciente a la *Universidade de São Paulo* (USP). Esto se hizo con el fin de cumplir con los requisitos establecidos en la Resolución 466/2012 del Consejo Nacional de Salud, que establece las directrices para la realización de estudios que involucren a seres humanos. Consecuentemente, el proyecto fue aprobado conforme al dictamen número 3.506.984/2019.

El investigador principal estuvo a cargo de la recolección de datos, asegurando la preservación del anonimato de los participantes y garantizando que toda la información se tratara de forma confidencial. Además, los resultados se presentaron de manera agregada, sin individualizar ni identificar a los participantes.

## Resultados

La investigación se llevó a cabo con 12 cursos de enfermería ofrecidos en siete Instituciones de Educación Superior, en los cuales 354 profesores eran elegibles para participar en el estudio y de estos, 179 respondieron a los cuestionarios (50,560%). Se excluyeron de la muestra a los profesionales con menos de seis meses de vínculo laboral. Entre los demás profesores invitados a participar en el estudio, tres manifestaron, en respuesta a la invitación por correo electrónico, incomodidad para evaluar a su coordinador(a), lo que los llevó a declinar la invitación a la investigación. En relación con los demás invitados, no hubo respuesta a la invitación realizada.

Entre los encuestados, la mayoría eran mujeres (86%), impartían asignaturas en el área de Ciencias de la Enfermería (83,2%), tenían un posgrado completado (94,4%) y eran empleados públicos (89,9%), conforme se muestra en la Tabla 1. Es importante destacar que las respuestas a los datos sociodemográficos en el formulario proporcionado no eran de carácter obligatorio. Como resultado, algunos ítems no fueron completados por todos los participantes de la investigación, lo que ocasionó ítems con menos de 179 respuestas.

**Tabla 1 -:** Presentación de los datos sociodemográficos de los profesores de los cursos de pregrado en enfermería (N = 179). Centro-Oeste, Brasil, 2021

	**Categorías**	**Tipo N (%)**
Edad	20 a 39 años	96 (53,630)
	40 a 69 años	79 (44,130)
	Total	175 (97,760)
Sexo	Masculino	25 (13,970)
	Femenino	154 (86,030)
	Total	179 (100)
Asignaturas	Ciencias Biológicas y de la Salud y Ciencias Humanas y Sociales	29 (16,200)
	Ciencias de la Enfermería	149 (83,240)
	Total	178 (99,440)
Grado de formación	Grado y/o especialización	10 (5,590)
	Maestría y/o doctorado	169 (94,410)
	Total	179 (100)
Tipo de relación laboral	Empleado público	161 (89,940)
	Contratado y/o voluntario	18 (10,060)
	Total	179 (100)
Jornada de trabajo	20, 30 o 40 horas	27 (15,090)
	40 horas con dedicación exclusiva	152 (84,910)
	Total	179 (100)
Años de docencia en el curso de enfermería	01 a 09 años	73 (40,780)
	10 a 19 años	77 (43,020)
	20 a 39 años	29 (16,200)
	Total	179 (100)
Ingreso salarial*	1 a 9 salarios mínimos^†^	88 (49,160)
	9,1 a 12 salarios mínimos^‡^	47 (26,260)
	12,1 a 15 salarios mínimos o más^§^	44 (24,580)
	Total	179 (100)
Ha ocupado un cargo de gestión	No	93 (51,960)
	Sí	86 (48,040)
	Total	179 (100)

* Salario mínimo R$ 1.100,00, Brasil, 2021; ^†^Salario entre R$ 1.100,00 a R$ 9.900,00; ^‡^Salario entre R$ 10.010,00 a R$ 13.200,00; ^§^Salario entre R$ 13.310,00 a R$ 16.500,00 o más

La forma en que los profesores comprenden el liderazgo auténtico de los coordinadores fue evaluada (Tabla 2). Con el objetivo de facilitar el análisis de las puntuaciones, los participantes fueron categorizados en tres niveles (bajo, medio y alto) en función del valor total de la puntuación, considerando que había un gran número de sujetos (179). Es importante señalar que el cuestionario tiene una diferencia en el número de preguntas entre los dominios. Así, la versión *RATER* podía alcanzar una puntuación máxima de 52 puntos. El análisis reveló que la mayoría de los profesores (72,000%) consideraron que sus coordinadores poseían un nivel alto de liderazgo auténtico en la administración académica del curso, con una mediana de 42 puntos. Solo el 3,300% de los profesores clasificaron a sus coordinadores como poseedores de un bajo liderazgo auténtico.

**Tabla 2 -:** Percepción de los profesores sobre el liderazgo auténtico de los coordinadores (N = 179). Centro-Oeste, Brasil, 2021

	**Puntaje**	**n**	**%**	**Mediana**
Relacional y Moral	0 – 9*	6	3,400	23
	10 – 18^†^	40	22,300	
	19 – 28^‡^	133	74,300	
Procesamiento Equilibrado de Información	0 – 4*	13	7,300	10
	5 – 8^†^	44	24,500	
	9 – 12^‡^	122	68,200	
Autoconciencia	0 – 4*	11	6,200	9
	5 – 8^†^	56	31,200	
	9 – 12^‡^	112	62,600	
Liderazgo Auténtico Total	0 – 17*	6	3,300	42
	18 – 34^†^	44	24,700	
	35 – 52^‡^	129	72,000	

*Bajo puntaje para el liderazgo auténtico; ^†^Medio puntaje para el liderazgo auténtico; ^‡^Alto puntaje para el liderazgo auténtico

Al analizar la correlación existente entre los factores de la satisfacción en el trabajo y las áreas o dimensiones del liderazgo auténtico, se observa que en el dominio Relacional y Moral hubo una correlación positiva con todos los elementos de la satisfacción en el trabajo, siendo la mayor correlación encontrada entre Relacional y Moral y la satisfacción con las relaciones jerárquicas (SRJ) (*rho* = 0,416, p < 0,0001). El Procesamiento Equilibrado de Información presentó una correlación significativa solo con la SRJ (*rho* = 0,335, p < 0,0001) y con la satisfacción laboral total (*rho* = 0,235, p = 0,002). En cuanto al dominio Autoconciencia, se observó una correlación positiva significativa entre SRJ (*rho* = 0,366, p < 0,0001), satisfacción intrínseca al trabajo (SIT) (*rho* = 0,215, p = 0,004) y satisfacción laboral total (*rho* = 0,292, p < 0,0001). Finalmente, al investigar el liderazgo auténtico total, se percibe que existe una correlación significativa con todos los indicadores de satisfacción en el trabajo, siendo la mayor de ellas con el factor SRJ (*rho* = 0,402, p < 0,0001) (Tabla 3).

**Tabla 3 -:** Correlación entre satisfacción laboral y liderazgo auténtico (N = 179). Centro-Oeste, Brasil, 2021

**Variables**	**Satisfacción en el trabajo**							
	SRJ*		**SAFT** ^†^		**SIT** ^‡^		**TOTAL**	
	***rho***	**p-valor**	***rho***	**p-valor**	***rho***	**p-valor**	***rho***	**p-valor**
Relacional y Moral	0,416^§^	<0,0001	0,210^||^	0,005	0,257^§^	0,001	0,365^§^	<0,0001
Procesamiento Equilibrado de Información	0,335^§^	<0,0001	0,126	0,093	0,110	0,142	0,235^||^	0,002
Autoconciencia	0,366^§^	<0,0001	0,127	0,089	0,215^||^	0,004	0,292^§^	<0,0001
Liderazgo Auténtico Total	0,402^§^	<0,0001	0,176^||^	0,018	0,226^||^	0,002	0,331^§^	<0,0001

*SRJ = Satisfacción con relaciones jerárquicas; ^†^SAFT = Satisfacción con el ambiente físico de trabajo; ^‡^SIT = Satisfacción intrínseca del trabajo; ^§^Correlación de Spearman significativa al nivel del 1%; ^||^ Correlación de Spearman significativa al nivel del 5%

A partir de las correlaciones encontradas entre liderazgo auténtico y satisfacción en el trabajo, se puede inferir que, según la percepción de los profesores, la adopción de prácticas de liderazgo auténtico en la administración de los cursos de pregrado está relacionada con una mejora en la satisfacción laboral docente.

La regresión logística binaria se ajustó para analizar la relación entre la variable dependiente Satisfacción en el trabajo y las variables independientes (dominios del liderazgo auténtico). El modelo mostró un buen ajuste, con un parámetro de calidad de ajuste estadísticamente significativo [Prueba de Hosmer y Lemeshow: X²(2) = 0,124, p = 0,940, R² de Nagelkerke = 0,130 y VIF = 1,140]. El modelo produjo coeficientes Beta (β) que indican la influencia de cada dominio del liderazgo auténtico en la satisfacción en el trabajo, siendo estos: Relacional y Moral (β = 1,700), Procesamiento Equilibrado de Información (β = 0,270), Autoconciencia (β = 0,060) y liderazgo auténtico total (β = 0,480) (como se ilustra en la Tabla 4).

**Tabla 4 -:** Razones de probabilidad entre liderazgo auténtico y satisfacción en el trabajo (N = 179). Centro-Oeste, Brasil, 2021

**Variables**		**Satisfacción en el trabajo**		B*	**Razón de Probabilidad (95%)**	**p-valor**
		**Insatisfecho**	**Satisfecho**			
		**N(%)**	**N(%)**			
Relacional y Moral	No	21(33,900)	10(8,500)	1,700	Referencia	<0,0001^†^
	Sí	41(66,100)	107(91,500)		5,480 (2,380 - 12,630)	
Procesamiento equilibrado de informaciones	No	22(35,500)	20(17,100)	0,270	Referencia	0,961
	Sí	40(64,500)	97(82,900)		1,030 (0,340 - 3,070)	
Autoconciencia	No	27(43,500)	24(20,500)	0,060	Referencia	0,926
	Sí	35(56,500)	93(79,500)		1,060 (0,310 - 3,570)	
Liderazgo auténtico total	No	24(38,700)	15(12,800)	0,480	Referencia	0,521
	Sí	38(61,300)	102(87,200)		1,610 (0,370 - 7,070)	

*Coeficiente Beta; ^†^Significativa para el nivel p ≤ 0,001

Al ejecutar el modelo de regresión logística binaria, se observó que solo el dominio Relacional y moral presentó significancia estadística (p-valor < 0,0001), indicando que cuando el dominio Relacional y moral está presente, la probabilidad de que los profesores estén satisfechos con su trabajo aumenta 5,480 veces. Al analizar la distribución de los profesores en el dominio Relacional y moral con la satisfacción laboral, se percibe que los altos índices de esta variable se encuentran en el grupo de satisfacción (n=107; 91,500%).

## Discusión

A partir de los datos sociodemográficos de los profesores participantes en la investigación, se constató que la mayoría tiene entre 20 y 39 años, es de sexo femenino, imparte clases en el área de las Ciencias de la Enfermería, posee un título de maestría y/o doctorado, es empleada pública, cumple una jornada laboral de 40 horas con dedicación exclusiva (DE), lleva trabajando entre 10 y 19 años en la enseñanza superior, tiene un ingreso salarial de 1 a 12 salarios mínimos y nunca ha ocupado cargos de gestión.

Del análisis de la relación entre el ejercicio del liderazgo auténtico por parte de los coordinadores y la satisfacción laboral de los profesores de los cursos de pregrado en enfermería de universidades públicas federales, se constató que los profesores involucrados en la investigación otorgaron las calificaciones más altas de la escala (“muchas veces, casi siempre”) a todos los dominios del liderazgo auténtico, así como a la evaluación global del liderazgo auténtico. Estos resultados sugieren que los participantes tuvieron una percepción altamente positiva del liderazgo auténtico. Esta conclusión está respaldada por investigaciones previas que también destacaron la prevalencia de líderes con niveles sustanciales de autenticidad en diversos dominios del liderazgo auténtico^(17-20)^.

Además, es importante señalar que los subordinados que perciben autenticidad en sus líderes tienden a tener mayor esperanza, optimismo, confianza y resiliencia^(18)^.

El liderazgo auténtico en la educación es visto como altamente positivo en la presente investigación; los resultados indican que los profesores consideraron elevadas las medidas de autenticidad en todos los dominios, lo que sugiere que los coordinadores poseen actitudes de un líder genuinamente auténtico. Esto es relevante para el contexto educativo, ya que indica que este modelo de liderazgo es valorado en dicho entorno, y los líderes que emplean el liderazgo auténtico pueden tener seguidores más comprometidos con la implementación de los cambios necesarios.

El liderazgo auténtico desempeña un papel fundamental en el contexto educativo al fomentar comportamientos positivos en los profesores y crear un ambiente laboral caracterizado por la confianza y el optimismo. Los líderes auténticos tienen la capacidad de influir en el entorno educativo, mejorando la eficacia de los profesores y ayudándolos a manejar diferentes situaciones, lo que refuerza el compromiso y reduce la insatisfacción^(19)^.

La práctica del liderazgo auténtico establece un entorno laboral propicio que promueve la autonomía de los subordinados e impulsa la motivación, especialmente cuando las decisiones del líder son justas y adecuadas para la institución^(20)^. Es evidente que la toma de decisiones basada en principios éticos es un componente clave del desempeño de los líderes en el contexto educativo, involucrando principios de justicia, igualdad y democracia. Por lo tanto, la promoción del liderazgo auténtico puede ser una estrategia importante para mejorar el entorno educativo, la satisfacción de los profesores y, en consecuencia, la calidad de la enseñanza^(2)^.

Al evaluar la relación entre el liderazgo auténtico y la satisfacción laboral docente, se observa que la presencia de liderazgo auténtico por parte de los coordinadores resulta en una mayor satisfacción por parte de los profesores. Esta relación también se ha verificado en otras áreas, como en la enfermería, donde los enfermeros clasifican a sus directores con un alto nivel de liderazgo auténtico, lo que resulta en una menor fatiga emocional y una mayor satisfacción laboral^(21)^.

En el ámbito de la educación, existe una correlación positiva entre la autenticidad del director y la confianza y el compromiso de los profesores^(22)^. Otra investigación encontró que el liderazgo auténtico y la satisfacción laboral de los empleados públicos están positivamente correlacionados con la motivación, y que estos factores tienen diferentes efectos en el compromiso de estos profesionales^(23)^. Además, un estudio indicó una correlación moderada y significativa de forma negativa entre el liderazgo auténtico y la presión laboral de los profesores, es decir, cuanto más auténtico es el líder, menor es el nivel de estrés percibido por los subordinados^(24)^.

Por lo tanto, al adoptar el modelo teórico del liderazgo auténtico, es posible establecer una relación transparente con los profesores, lo que puede mejorar la motivación, la satisfacción y la confianza mutua. Esto crea un entorno de seguridad y respeto, fortaleciendo la ética y moral de los profesionales, y resultando en una mayor percepción de los profesores sobre el apoyo organizacional^(25)^.

Con base en los datos presentados, es evidente que los modelos contemporáneos de liderazgo están relacionados con la satisfacción laboral, aunque de forma relativa. Los resultados indican que el liderazgo auténtico impacta directamente en la satisfacción laboral de los profesores de enfermería desde diferentes perspectivas. Sin embargo, los líderes deben ser conscientes de que la organización no siempre podrá satisfacer todas las expectativas de los subordinados, por lo que será necesario esforzarse para ofrecer un ambiente laboral confiable, flexible y que promueva relaciones laborales positivas.

Se observó que el liderazgo auténtico explica solo el 13,500% de la variación total en la satisfacción laboral, lo cual es considerado representativo para un modelo teórico relativamente nuevo, como el liderazgo auténtico. Después del análisis, es posible afirmar que existe una asociación significativa entre el liderazgo auténtico y la satisfacción laboral, especialmente en el dominio Relacional y Moral, ya que aumenta en 5,48 veces la probabilidad de sentirse satisfecho en el ejercicio profesional. Aunque los otros dominios del liderazgo auténtico no presentaron significancia estadística, aún muestran una relación positiva con la satisfacción laboral de los profesores de enfermería en las universidades federales de la región Centro-Oeste de Brasil.

Otros estudios recientes también han destacado los mismos dominios del liderazgo auténtico, lo que confirma la importancia de estos factores para el constructo^(22,24,26-28)^. Sin embargo, algunos estudios han presentado divergencias en la evaluación de los dominios del liderazgo auténtico. Por ejemplo, un estudio realizado con enfermeros recién graduados observó que la autoconciencia del líder mostró una correlación débil con el compromiso laboral^(29)^. En otro estudio realizado en escuelas de Pakistán, hubo variación en la evaluación de los dominios por parte de los profesores, con la mayoría clasificando al director como Relacional y Moral, pero en algunas escuelas, el director fue evaluado como teniendo un Procesamiento Equilibrado de Información o Autoconciencia^(28)^. No obstante, un estudio con líderes académicos mostró consistencia en todas las dimensiones del liderazgo auténtico, excepto en el dominio Relacional y Moral^(27)^.

Aunque la literatura sobre educación democrática no utiliza los mismos términos del liderazgo auténtico, como Relacional y Moral, Procesamiento Equilibrado de Información y Autoconciencia, los conceptos subyacentes a estos dominios, como la cultura de la honestidad, el compromiso y la toma de decisiones participativa, son componentes esenciales en el entorno educativo democrático^(27)^.

Con base en los resultados presentados, se concluye que el modelo teórico del Liderazgo Auténtico es altamente deseable y eficaz para el desarrollo del factor humano, generando resultados positivos en las organizaciones, incluidas las instituciones educativas. Según la percepción de los profesores, los tres dominios del Liderazgo Auténtico fueron considerados importantes para los cursos de Enfermería en las universidades federales. Por lo tanto, la adopción de este modelo teórico es vista como importante para el contexto educativo.

Este estudio presentó algunas limitaciones, como el uso exclusivo de encuestas en línea y el muestreo por conveniencia. Debido a la heterogeneidad de la muestra, los resultados no pueden generalizarse a los profesores que trabajan en universidades públicas federales de otras regiones de Brasil. Por lo tanto, es necesario realizar investigaciones nacionales con muestras más amplias para validar los resultados encontrados.

## Conclusión

Los resultados del estudio indicaron una asociación positiva significativa, mostrando que la satisfacción laboral de los profesores en los cursos de pregrado en enfermería está influenciada por la práctica del liderazgo auténtico por parte de los coordinadores, destacándose tres variables: Relacional y Moral, Procesamiento Equilibrado de Información y Autoconciencia. Esto confirma la hipótesis planteada en la investigación y sugiere que los comportamientos auténticos de liderazgo pueden influir positivamente en el entorno laboral docente. Esta comprensión puede llevar a una mejora en la gestión mediante decisiones más acertadas en el ámbito educativo.

El estudio realizado destaca la importancia del liderazgo auténtico como un factor significativo en la satisfacción laboral de los profesores de pregrado en enfermería en las universidades públicas federales de la región *Centro-Oeste* de Brasil. Aunque la asociación es modesta, el liderazgo auténtico, a pesar de ser un modelo teórico relativamente reciente, tiene una representatividad significativa.

El dominio Relacional y Moral mostró una relación significativa con la satisfacción laboral de los profesores, lo que refuerza la importancia de estos factores para el constructo. Sin embargo, es importante recordar que hay otros factores que influyen en la satisfacción laboral y que deben ser investigados en estudios futuros. El liderazgo auténtico establece una base sólida para la adopción de modelos de liderazgo contemporáneos e innovadores en el entorno educativo, y la orientación e integración continuas son fundamentales para garantizar un ambiente laboral positivo y satisfactorio.

Los resultados obtenidos confirman la relevancia del liderazgo auténtico en el ámbito educativo, especialmente en el contexto de la educación en enfermería, y abren nuevas oportunidades para futuras investigaciones sobre modelos de gestión y liderazgo en el área educativa. Además, estos hallazgos proporcionan orientaciones valiosas para coordinadores, gestores y líderes educativos, permitiendo que basen sus prácticas de gestión y liderazgo en los principios del liderazgo auténtico. Esto, a su vez, contribuye a la promoción de relaciones laborales más positivas y al aumento de los niveles de satisfacción en el entorno laboral.

# Translation: Relação entre liderança autêntica e satisfação no trabalho de professores de graduação em enfermagem*

**DOI:** 10.1590/1518-8345.7292.4477

**Authors:** Cezar Augusto da Silva Flores, André Almeida de Moura, José Luis Guedes dos Santos, Alexandre Pazetto Balsanelli, Carmen Silvia Gabriel, Andrea Bernardes

**Affiliations:** 1 Universidade Federal de Mato Grosso, Instituto de Ciências da Saúde, Sinop, MT, Brasil; 2 Universidade Federal de Minas Gerais, Escola de Enfermagem, Belo Horizonte, MG, Brasil; 3 Universidade Federal de Santa Catarina, Departamento de Enfermagem, Florianópolis, SC, Brasil; 4 Universidade Federal de São Paulo, Departamento de Administração em Serviço de Saúde e Enfermagem, São Paulo, SP, Brasil; 5 Universidade de São Paulo, Escola de Enfermagem de Ribeirão Preto, PAHO/WHO Collaborating Centre for Nursing Research Development, Ribeirão Preto, SP, Brasil

**Keywords:** Liderança, Satisfação no Trabalho, Enfermagem, Educação, Educação em Enfermagem, Docentes de Enfermagem

## Abstract

analisar a relação entre o exercício da liderança autêntica pelos coordenadores e a satisfação no trabalho de professores dos cursos de graduação em enfermagem de universidades públicas federais.

trata-se de um estudo de abordagem quantitativa, com delineamento observacional analítico e transversal. Doze cursos de graduação em enfermagem em Instituições de Ensino Superior públicas foram escolhidos para participar do estudo. As informações coletadas foram analisadas utilizando técnicas de estatística descritiva, teste de correlação de Spearman e análise de regressão logística binária, com um nível de significância de 5%.

a amostra consistiu-se de 179 professores que preencheram o Questionário Sociodemográfico, o Questionário de Liderança Autêntica e o Questionário de Satisfação no Trabalho. Os professores avaliaram que os coordenadores dos cursos apresentaram altos níveis de prática de liderança autêntica. Quanto à regressão logística binária, percebeu-se que apenas o domínio Relacional e Moral apresentou significância (p-valor < 0,0001), indicando que, quando este domínio está presente, há um aumento em 5,48 vezes a chance de os professores estarem satisfeitos.

os resultados do estudo indicaram que a satisfação no trabalho dos professores nos cursos de graduação em enfermagem é influenciada pela prática da liderança autêntica por parte dos coordenadores.

## Destaques

**(1)** Avanços na prática da liderança autêntica na gestão educacional.

**(2)** Os instrumentos utilizados são úteis para avaliar a liderança e a satisfação.

**(3)** Identificadas relações entre a liderança autêntica e a satisfação no trabalho.

**(4)** Mensurar a liderança autêntica e a satisfação no trabalho no ambiente educacional.

## Introdução

Nos últimos anos, mudanças políticas, sociais, econômicas e culturais transformaram as relações de trabalho, destacando a necessidade de desenvolver resiliência, eficácia e otimismo nos funcionários. Nesse contexto, capacitar profissionais com flexibilidade e aprimoramento de habilidades, especialmente em liderança, torna-se essencial para romper com modelos de gestão ultrapassados^(1-4)^.

A liderança autêntica emerge como um modelo teórico que enfatiza o caráter moral e ético do líder, promovendo transparência nas relações de trabalho e gerando respeito e confiança por meio de comportamentos alinhados aos valores e convicções pessoais. Essa abordagem foca em estabelecer relações positivas entre líderes e liderados, fundamentadas na fidelidade do líder às suas necessidades, desejos, crenças e emoções^(2,5-6)^.

O modelo teórico da liderança autêntica caracteriza-se por comunicação transparente, autoconsciência e equilíbrio na tomada de decisões, priorizando interesses coletivos e o desenvolvimento dos colaboradores, com potencial para influenciar positivamente o ambiente de trabalho, especialmente no bem-estar, no desempenho e no fator psicológico dos seguidores^(2,5-6)^.

Já a satisfação no trabalho é a percepção do profissional sobre a relação entre suas expectativas e o que obtém no ambiente laboral, resultando em prazer emocional. Há um consenso de que a satisfação no trabalho influencia a satisfação geral, pois emoções e atitudes no trabalho afetam a vida pessoal^(7-8)^.

No contexto educacional, pesquisas sobre satisfação no trabalho são escassas e muitas vezes incorporam teorias e conceitos de outras áreas, desconsiderando as particularidades desse ambiente^(9)^. Apesar da escassez de pesquisas sobre satisfação no trabalho no contexto educacional, estudos existentes indicam que as relações sociais no ambiente laboral são mais influentes na satisfação do que recompensas salariais^(2,9-10)^. A relação entre liderança autêntica e satisfação no trabalho é bem documentada em diversas áreas, mas faltam evidências específicas sobre professores de enfermagem^(2)^.

Diante do exposto, este estudo se justifica ao explorar minuciosamente a relação entre a aplicação do conceito de liderança autêntica pelos coordenadores de cursos de graduação e seu impacto na satisfação no trabalho dos professores dos cursos de enfermagem. Importante ressaltar que não foram identificados estudos que abordassem a temática, tornando este estudo pioneiro.

Partindo da hipótese de que a liderança autêntica exercida pelos coordenadores dos cursos de graduação em enfermagem apresenta associação com a satisfação no trabalho de docentes, esta pesquisa tem o potencial de revelar informações ainda não exploradas pela comunidade científica, com a ressalva de que seus resultados podem orientar instituições de ensino na promoção do crescimento profissional, baseando-se em padrões acadêmicos elevados.

Dessa forma, o presente estudo teve como objetivo analisar a relação entre o exercício da liderança autêntica pelos coordenadores e a satisfação no trabalho de professores dos cursos de graduação em enfermagem de universidades públicas federais.

## Método

### Tipo do estudo

Trata-se de um estudo de abordagem quantitativa, com delineamento observacional analítico e transversal. Desse modo, na presente pesquisa, utilizou-se o guia de recomendações *Strengthening the Reporting of Observational Studies in Epidemiology* (STROBE) como ferramenta para subsidiar a comunicação dos resultados evidenciados^(11)^.

### Local do estudo

A investigação ocorreu nos programas de graduação em enfermagem de instituições federais de ensino superior situadas na região Centro-Oeste do Brasil, abrangendo os estados de Goiás, Mato Grosso e Mato Grosso do Sul e o Distrito Federal (Brasília).

Esta região foi selecionada devido à escassez de investigações científicas no campo da gestão acadêmica e da satisfação no ambiente de trabalho, especialmente no contexto acadêmico. Além disso, esta região cobre uma ampla extensão territorial no Brasil, com poucos grupos de pesquisa que se dedicam à produção de conhecimento na área da gestão e liderança em enfermagem.

### População do estudo

A população do estudo foi definida a partir de uma amostra, composta pelos professores que atuavam nos cursos de graduação em enfermagem dessas universidades, considerando-se o critério de inclusão: ser docente com vínculo institucional efetivo ou interino com as referidas universidades e contar com um coordenador que possua, no mínimo, seis meses de experiência na gestão acadêmica do curso.

Realizou-se o cálculo do tamanho da amostra para uma população finita. Dado que a população elegível para o estudo consistia de 354 professores, estimou-se o tamanho da amostra em 184 participantes, com margem de erro de 5% e nível de confiança de 95%. Entretanto, a pesquisa abrangeu 179 professores de 12 cursos de Enfermagem oferecidos por sete Instituições de Ensino Superior que responderam aos questionários. Em decorrência do não cumprimento total do tamanho da amostra, foi recalculada a margem de erro para o número de respostas coletadas (179), sendo esta de 5,1%, com um nível de confiança de 95%. Ratifica-se que a diminuição da amostra não causou perdas ao resultado da pesquisa.

### Coleta de dados e período

A coleta de dados teve início no mês de agosto e término no mês de novembro de 2021 e transcorreu por meio do *Google Forms* ^®^, que foi disponibilizado por meio de links eletrônicos ou aplicativos de mensagens. Os participantes receberam um prazo de sete dias para responder aos questionários após o envio do convite. Em caso de não resposta, foram enviados lembretes com um intervalo de sete dias até a quinta tentativa.

Os participantes receberam convites por e-mail ou mensagem com *links* para uma plataforma privada, onde tiveram que preencher o Termo de Consentimento Livre e Esclarecido (TCLE) antes de acessar os questionários da pesquisa.

### Instrumentos para a coleta de dados

Primeiramente, foi elaborado, pelo pesquisador, um questionário sociodemográfico para obtenção das informações sobre a caracterização dos sujeitos da pesquisa, o que contribuiu para contextualizar os resultados.

Para avaliar a autenticidade dos líderes, os participantes do estudo foram convidados a preencher o Questionário de Liderança Autêntica (ALQ)^(12)^, validado por pesquisadores no Brasil^(13)^. O ALQ possui duas versões: a primeira, intitulada “Avaliando meu Jeito de Liderar” (SELF), em que os líderes realizam uma autoavaliação do seu nível de liderança autêntica; e a segunda, denominada “Avaliando o seu Líder” (RATER), em que os liderados avaliam o grau de liderança autêntica dos seus líderes^(12-13)^, sendo esta a versão utilizada na presente pesquisa.

Depois que o instrumento foi validado para uso na língua portuguesa do Brasil, houve a adaptação das duas versões (SELF e RATER), sendo que a versão RATER foi reduzida de 16 para 13 itens distribuídos em três domínios: Relacional e Moral, Processamento Equilibrado de Informação e Autoconsciência^(13)^.

Compreende-se por Relacional e Moral quando o líder se esforça para estabelecer relacionamentos transparentes e sinceros, baseados em valores morais e éticos sólidos, visando o bem-estar e o crescimento de seus liderados. O Processamento Equilibrado de Informação envolve a análise objetiva de todas as informações relevantes antes que o líder tome uma decisão. Essa abordagem busca considerar diversos aspectos e pontos de vista, garantindo uma tomada de decisão embasada e imparcial. A Autoconsciência refere-se à capacidade do líder em reconhecer-se como um ser humano, possuindo discernimento sobre suas habilidades e limitações^(12-13)^.

Cada item é avaliado em uma escala ordinal de frequência, variando de zero a quatro. Para cada item, o participante escolhe uma das cinco opções em uma escala Likert, cujos valores de 0 a 4 representam o grau de autenticidade do líder, sendo: 0 = raramente/nunca; 1 = de vez em quando; 2 = às vezes; 3 = frequentemente; 4 = muitas vezes/quase sempre. Dessa forma, escores mais altos indicam uma maior percepção de autenticidade, tanto por parte do líder quanto do liderado^(12-13)^.

Para coletar informações sobre a satisfação no trabalho, utilizou-se o Questionário de Satisfação no Trabalho (S20/23) adaptado para o português do Brasil^(14)^. O questionário consiste em 20 itens divididos em três categorias/fatores e foi avaliado utilizando-se uma escala do tipo Likert de cinco pontos, na qual 1 indica totalmente insatisfeito e 5 indica totalmente satisfeito. A fim de avaliar a satisfação global, a mediana foi calculada para cada um dos fatores, considerando-se os fatores que apresentaram mediana superior a três pontos indicavam alta tendência de satisfação^(14)^.

### Tratamento e análise dos dados

Foi utilizado o *softwareIBM Statistical Package for the Social Sciences* (SPSS), versão 22 em português, para tratar os dados coletados. Na análise estatística foram conduzidas análises descritivas e inferenciais para avaliar as relações entre as variáveis.

Inicialmente, realizou-se um levantamento descritivo para avaliar a percepção dos professores em relação à liderança autêntica, utilizando a versão RATER do questionário e considerando seus domínios (Relacional e Moral, Processamento Equilibrado de Informações e Autoconsciência), bem como a liderança autêntica total. Os dados foram submetidos ao teste de normalidade de Kolmogorov-Smirnov com a correção de Lilliefors e, ao se perceber que os mesmos não seguiam distribuição normal, os resultados foram expressos em termos de posição, utilizando escores e medianas.

O teste de correlação de Spearman (*rho*) foi usado para investigar a relação entre a liderança autêntica (ALQ) e a satisfação no trabalho, juntamente com seus domínios e fatores associados. Essa é uma técnica estatística não paramétrica adequada para avaliar a relação entre variáveis ordinais ou quando os dados não têm distribuição normal^(15)^.

Ao examinar as relações entre os componentes da satisfação no trabalho e os diferentes aspectos da liderança autêntica, optou-se por conduzir a análise de regressão logística binomial.

A análise de regressão logística binomial permite estimar a probabilidade de um resultado categórico específico (satisfação no trabalho) com base em um ou mais fatores preditivos (liderança autêntica), que podem ser categóricos ou contínuos^(16)^. Antes de se aplicar o modelo, foi avaliada a multicolinearidade entre as variáveis por meio do Fator de Inflação de Variância (VIF)^(16)^.

Para esse propósito, todas as variáveis foram discretizadas em categorias dicotômicas, com o intuito de quantificar as razões de chances associadas à satisfação no trabalho em relação a cada domínio da liderança autêntica. Realizou-se essa conversão dos dados multinominais para binomiais, uma vez que os dados originais apresentavam considerável dispersão. Dessa maneira, os dados foram agrupados em duas categorias distintas: “não ou ausente”, representada pelo valor zero (0), e “sim ou presente”, que recebeu o valor um (1). Esse procedimento tornou a análise mais sólida e robusta.

A qualidade do ajuste foi verificada pelo teste Hosmer e Lemeshow, que mede o grau de acurácia do modelo logístico e, por fim, o R2 Nagelkerke (ajustado), que indica o quanto da variação no resultado pode ser explicada pela variável preditora (isto é, a liderança autêntica). Quanto maior o R2, melhor o ajuste do modelo^(16)^.

### Aspectos éticos

Observando os princípios éticos que regem a pesquisa com participantes humanos, o projeto foi encaminhado à análise do Comitê de Ética em Pesquisa da Escola de Enfermagem de Ribeirão Preto, pertencente à Universidade de São Paulo (USP). Isso foi feito com o intuito de atender aos requisitos estabelecidos na Resolução 466/2012 do Conselho Nacional de Saúde, que estabelece as diretrizes para a condução de estudos envolvendo seres humanos. Consequentemente, o projeto recebeu aprovação, conforme parecer número 3.506.984/2019.

O pesquisador principal foi encarregado pela coleta de dados, sendo assegurada a preservação do anonimato dos participantes, garantindo que todas as informações fossem tratadas de forma confidencial. Além disso, os resultados foram apresentados de maneira agregada, sem nunca individualizar ou identificar os participantes.

## Resultados

A pesquisa foi realizada com 12 cursos de enfermagem ofertados em sete Instituições de Ensino Superior, sendo que 354 professores estavam aptos a participar do estudo. Destes, 179 responderam aos questionários (50,560%). Foram excluídos da amostra os profissionais com menos de seis meses de vínculo empregatício. Dentre os demais professores convidados a participar do estudo, três manifestaram, em resposta ao convite por *e-mail*, desconforto em avaliar o(a) seu(sua) coordenador(a), o que os levou a declinar do convite para a pesquisa. Em relação aos demais convidados, não houve retorno ao convite realizado.

Dentre os respondentes, a maioria era do sexo feminino (86%), ministravam disciplinas na área das Ciências de Enfermagem (83,2%), apresentavam conclusão da pós-graduação (94,4%) e eram c oncursados (89,9%), conforme consta na Tabela 1. É relevante salientar que as respostas aos dados sociodemográficos no formulário disponibilizado não eram de caráter obrigatório. Como resultado, alguns itens não foram preenchidos por todos os participantes da pesquisa, ocasionando itens com menos de 179 respondentes.

**Tabela 1 -:** Apresentação dos dados sociodemográficos dos professores dos cursos de graduação em enfermagem (N = 179). Centro-Oeste, Brasil, 2021

	**Categorias**	**Tipo N (%)**
Idade	20 a 39 anos	96 (53,630)
	40 a 69 anos	79 (44,130)
	Total	175 (97,760)
Sexo	Masculino	25 (13,970)
	Feminino	154 (86,030)
	Total	179 (100)
Disciplinas	Ciências Biológicas e da Saúde e Ciências Humanas e Sociais	29 (16,200)
	Ciências da Enfermagem	149 (83,240)
	Total	178 (99,440)
Grau de formação	Graduação e/ou especialização	10 (5,590)
	Mestrado e/ou doutorado	169 (94,410)
	Total	179 (100)
Tipo de vínculo empregatício	Concursado	161 (89,940)
	Contratado e/ou voluntário	18 (10,060)
	Total	179 (100)
Jornada de trabalho	20, 30 ou 40 horas	27 (15,090)
	40 horas com dedicação exclusiva	152 (84,910)
	Total	179 (100)
Anos de magistério no curso de enfermagem	01 a 09 anos	73 (40,780)
	10 a 19 anos	77 (43,020)
	20 a 39 anos	29 (16,200)
	Total	179 (100)
Renda salarial*	1 a 9 salários-mínimos^†^	88 (49,160)
	9,1 a 12 salários-mínimos^‡^	47 (26,260)
	12,1 a 15 salários-mínimos ou mais^§^	44 (24,580)
	Total	179 (100)
Já assumiu cargo de gestão	Não	93 (51,960)
	Sim	86 (48,040)
	Total	179 (100)

*Salário-mínimo R$ 1.100,00, Brasil, 2021; ^†^Salário entre R$ 1.100,00 a R$ 9.900,00; ^‡^Salário entre R$ 10.010,00 a R$ 13.200,00; ^§^Salário entre R$ 13.310,00 a R$ 16.500,00 ou mais

A forma de compreensão dos professores em relação à liderança autêntica dos coordenadores foi avaliada (Tabela 2). Com o objetivo de facilitar a análise dos escores, os participantes foram categorizados em três níveis (baixa, média e alta) com base no valor total do escore, considerando que havia um grande número de sujeitos (179). É importante ressaltar que o questionário tem uma diferença no número de questões entre os domínios. Assim, a versão RATER poderia atingir um escore máxima de 52 pontos. A análise revelou que a maioria dos professores (72,000%) considerou que seus coordenadores possuíam um nível elevado de liderança autêntica na administração acadêmica do curso, apresentando mediana de 42 pontos. Apenas 3,300% dos professores classificaram seus coordenadores como tendo baixa liderança autêntica.

**Tabela 2 -:** Percepção dos professores em relação à liderança autêntica dos coordenadores (N = 179). Centro-Oeste, Brasil, 2021

	**Escore**	**n**	**%**	**Mediana**
Relacional e Moral	0 – 9*	6	3,400	23
	10 – 18^†^	40	22,300	
	19 – 28^‡^	133	74,300	
Processamento Equilibrado de Informações	0 – 4*	13	7,300	10
	5 – 8^†^	44	24,500	
	9 – 12^‡^	122	68,200	
Autoconsciência	0 – 4*	11	6,200	9
	5 – 8^†^	56	31,200	
	9 – 12^‡^	112	62,600	
Liderança Autêntica Total	0 – 17*	6	3,300	42
	18 – 34^†^	44	24,700	
	35 – 52^‡^	129	72,000	

*Baixo escore para a liderança autêntica; ^†^Médio escore para a liderança autêntica; ^‡^Alto escore para a liderança autêntica

Quando analisada a correlação existente entre os fatores da satisfação no trabalho e as áreas ou dimensões da liderança autêntica, percebe-se que, no domínio Relacional e Moral, houve correlação positiva com todos os elementos da satisfação no trabalho, sendo que a maior correlação encontrada foi entre o Relacional e Moral e a satisfação com as relações hierárquicas (SRH) (*rho* = 0,416, p < 0,0001). O Processamento Equilibrado de Informações apresentou correlação significante apenas com a SRH (*rho* = 0,335, p < 0,0001) e com satisfação no trabalho total (*rho* = 0,235, p = 0,002). Já no domínio Autoconsciência, percebeu-se correlação positiva significativa entre SRH (*rho* = 0,366, p < 0,0001), satisfação intrínseca ao trabalho (SIT) (*rho* = 0,215, p = 0,004) e satisfação no trabalho total (*rho* = 0,292, p < 0,0001). E, finalmente, ao investigar a liderança autêntica total, percebe-se que existe correlação significativa com todos os indicadores da satisfação no trabalho, sendo a maior delas com o fator SRH (*rho* = 0,402, p < 0,0001) (Tabela 3).

**Tabela 3 -:** Correlação entre satisfação no trabalho e a liderança autêntica (N=179). Centro-Oeste, Brasil, 2021

**Variáveis**	**Satisfação no trabalho**							
	SRH*		**SAFT** ^†^		**SIT** ^‡^		**TOTAL**	
	***rho***	**p-valor**	***rho***	**p-valor**	***rho***	**p-valor**	***rho***	**p-valor**
Relacional e Moral	0,416^§^	<0,0001	0,210^||^	0,005	0,257^§^	0,001	0,365^§^	<0,0001
Processamento Equilibrado de Informações	0,335^§^	<0,0001	0,126	0,093	0,110	0,142	0,235^||^	0,002
Autoconsciência	0,366^§^	<0,0001	0,127	0,089	0,215^||^	0,004	0,292^§^	<0,0001
Liderança Autêntica Total	0,402^§^	<0,0001	0,176^||^	0,018	0,226^||^	0,002	0,331^§^	<0,0001

*SRH = Satisfação com relações hierárquicas; ^†^SAFT = Satisfação com ambiente físico do trabalho; ^‡^SIT = Satisfação intrínseca do trabalho; ^§^Correlação de Spearman significante ao nível de 1%; ^||^Correlação de Spearman significante ao nível de 5%

A partir das correlações encontradas entre liderança autêntica e satisfação no trabalho, pode-se inferir que, conforme a percepção dos professores, a adoção de práticas da liderança autêntica na administração dos cursos de graduação está relacionada à melhora na satisfação com o trabalho docente.

A regressão logística binária foi ajustada para analisar a relação entre a variável dependente Satisfação no trabalho e as variáveis independentes (domínios da liderança autêntica). O modelo se mostrou bem ajustado, com parâmetro de qualidade do ajuste estatisticamente significante [Teste de Hosmer e Lemeshow: X^2^(2) = 0,124, p = 0,940, R^2^ Nagelkerke = 0,130 e VIF=1,140]. O modelo produziu coeficientes Beta (β) que indicam a influência de cada domínio da liderança autêntica na satisfação no trabalho, sendo eles: Relacional e Moral (β = 1,700), Processamento Equilibrado de Informações (β = 0,270), Autoconsciência (β = 0,060) e liderança autêntica total (β = 0,480) (conforme ilustrado na Tabela 4).

**Tabela 4 -:** Razões de chance entre liderança autêntica e satisfação no trabalho (N = 179). Centro-Oeste, Brasil, 2021

**Variáveis**		**Satisfação no trabalho**		B*	**Razão de Chance (95%)**	**p-valor**
		**Insatisfeito**	**Satisfeito**			
		**N(%)**	**N(%)**			
Relacional e Moral	Não	21(33,900)	10(8,500)	1,700	Referência	<0,0001^†^
	Sim	41(66,100)	107(91,500)		5,480 (2,380 - 12,630)	
Processamento equilibrado de informações	Não	22(35,500)	20(17,100)	0,270	Referência	0,961
	Sim	40(64,500)	97(82,900)		1,030 (0,340 - 3,070)	
Autoconsciência	Não	27(43,500)	24(20,500)	0,060	Referência	0,926
	Sim	35(56,500)	93(79,500)		1,060 (0,310 - 3,570)	
Liderança autêntica total	Não	24(38,700)	15(12,800)	0,480	Referência	0,521
	Sim	38(61,300)	102(87,200)		1,610 (0,370 - 7,070)	

*Coeficiente Beta; ^†^Significativa para o nível p ≤ 0,001

Ao rodar o modelo de regressão logística binária, percebeu-se que apenas o domínio Relacional e Moral apresentou significância estatística (p-valor < 0,0001), indicando que quando o domínio Relacional e Moral está presente, há aumento em 5,480 vezes a chance de os professores estarem satisfeitos com o trabalho. Ao analisar a distribuição dos professores no domínio Relacional e Moral com a satisfação no trabalho, percebe-se que os altos índices dessa variável estão entre o grupo de satisfação (n=107; 91,500%).

## Discussão

A partir dos dados sociodemográficos dos professores participantes da pesquisa, constatou-se que a maioria tem entre 20 e 39 anos, é do sexo feminino, ministra disciplina na área das ciências de enfermagem, possui título de mestre e/ou doutor, é concursada, cumpre jornada de trabalho de 40 horas com DE, está trabalhando de 10 a 19 anos no magistério superior, com uma renda salarial de 1 a 12 salários mínimos e nunca assumiu cargos de gestão.

A partir da análise da relação entre o exercício da liderança autêntica pelos coordenadores e a satisfação no trabalho dos professores dos cursos de graduação em enfermagem de universidades públicas federais, constatou-se que os professores envolvidos na pesquisa atribuíram as classificações mais elevadas da escala (“muitas vezes”, “quase sempre”) a todos os domínios da liderança autêntica, assim como à avaliação global da liderança autêntica. Esses resultados sugerem que os participantes tiveram uma percepção altamente positiva da liderança autêntica. Essa conclusão encontra respaldo em pesquisas anteriores que também destacaram a prevalência de líderes com níveis substanciais de autenticidade em diversos domínios da liderança autêntica^(17-20)^.

Além disso, é importante notar que os liderados que percebem autenticidade em seus líderes tendem a ter maior esperança, otimismo, confiança e resiliência^(18)^.

A liderança autêntica na educação é vista como altamente positiva na presente pesquisa; os resultados indicam que os professores consideraram como elevadas as medidas de autenticidade em todos os domínios, sugerindo que os coordenadores possuem atitudes de um líder genuinamente autêntico. Isso é relevante para o contexto educacional, pois indica que o modelo de liderança representa valorização neste ambiente e líderes que usam a liderança autêntica podem ter seguidores mais engajados na implementação das mudanças necessárias.

A liderança autêntica desempenha um papel fundamental no contexto educacional ao incentivar comportamentos positivos dos professores e fomentar um ambiente de trabalho caracterizado por confiança e otimismo. Os líderes autênticos têm a capacidade de influenciar o ambiente educacional, aumentando a eficácia dos professores e ajudando-os a lidar com diferentes situações, o que reforça o comprometimento e reduz a insatisfação^(19)^.

A prática da liderança autêntica estabelece um ambiente de trabalho propício que encoraja a autonomia dos liderados e impulsiona a motivação, especialmente quando as decisões do líder são justas e adequadas para a instituição^(20)^. É evidente que a tomada de decisões baseadas em princípios éticos é um componente fundamental da atuação dos líderes no contexto educacional, envolvendo princípios de justiça, igualdade e democracia. Portanto, a promoção da liderança autêntica pode ser uma importante estratégia para melhorar o ambiente educacional, a satisfação dos professores e, por consequência, a qualidade do ensino^(2)^.

Ao avaliar a relação entre a liderança autêntica e a satisfação no trabalho docente, observa-se que a presença de liderança autêntica por parte dos coordenadores resulta em maior satisfação por parte dos professores. Essa relação também é verificada em outras áreas, como na enfermagem, em que enfermeiros classificam seus diretores com alto nível de liderança autêntica, resultando em menor exaustão emocional e maior satisfação no trabalho^(21)^.

Na área da educação, existe correlação positiva entre a autenticidade do diretor e a confiança e engajamento dos professores^(22)^. Outra pesquisa constatou que a liderança autêntica e a satisfação no trabalho dos funcionários públicos estão correlacionadas de forma positiva com a motivação, e que esses fatores têm diferentes efeitos no engajamento desses profissionais^(23)^. Além disso, um estudo indicou uma correlação moderada e significativa de forma negativa entre a liderança autêntica e a pressão no trabalho dos professores, ou seja, quanto mais autêntico o líder, menor o nível de estresse percebido pelos liderados^(24)^.

Portanto, ao adotar o modelo teórico da liderança autêntica, é possível estabelecer relação transparente com os professores, podendo melhorar a motivação, a satisfação e a confiança mútua. Isso cria um ambiente de segurança e respeito, fortalecendo a ética e moral dos profissionais, resultando em maior percepção dos professores sobre o suporte organizacional^(25)^.

Com base nos dados apresentados, é evidente que os modelos contemporâneos de liderança estão relacionados à satisfação no trabalho, embora de forma relativa. Os resultados indicam que a liderança autêntica impacta diretamente na satisfação no trabalho dos enfermeiros professores sob diferentes perspectivas. Contudo, os líderes precisam ser sabedores de que nem sempre a organização será capaz de atender a todas as perspectivas dos liderados, sendo assim, é preciso esforçar-se para oferecer uma atmosfera de trabalho confiável e flexível, promovendo relações de trabalho positivas.

Observou-se que a liderança autêntica explica apenas 13,500% da variação total na satisfação no trabalho, o que é ponderado como representativo para um modelo teórico relativamente novo, a exemplo da própria noção de liderança autêntica. Após a análise, é possível afirmar que há associação expressiva entre a liderança autêntica e a satisfação no trabalho, especialmente no domínio Relacional e Moral, já que aumenta em 5,48 vezes a chance de se sentir satisfeito no exercício profissional. Embora os outros domínios da liderança autêntica não tenham apresentado significância estatística, ainda assim demonstram uma relação positiva com a satisfação no trabalho dos professores de enfermagem nas universidades federais do Centro-Oeste brasileiro.

Outros estudos recentes também destacaram os mesmos domínios da liderança autêntica, o que confirma a importância desses fatores para o constructo^(22,24,2022-28)^. No entanto, alguns estudos apresentaram divergências na avaliação dos domínios da liderança autêntica. Por exemplo, um estudo realizado com enfermeiros recém-formados, observou que a autoconsciência do líder apresentou correlação fraca com o envolvimento no trabalho^(29)^. Em outro estudo realizado em escolas no Paquistão, houve variação na avaliação dos domínios pelos professores, com a maioria classificando o diretor como Relacional e Moral, mas em algumas escolas, o diretor foi avaliado como tendo um Processamento Equilibrado de Informações ou Autoconsciência^(28)^. No entanto, um estudo com líderes acadêmicos mostrou consistência em todas as dimensões da liderança autêntica, exceto no domínio Relacional e Moral^(27)^.

Embora a literatura sobre educação democrática não utilize os mesmos termos da liderança autêntica, como Relacional e Moral, Processamento Equilibrado de Informações e Autoconsciência, os conceitos subjacentes a esses domínios, como a cultura da honestidade, o compromisso e a tomada de julgamento participativo, são componentes essenciais no ambiente educacional democrático^(27)^.

Com base nos resultados apresentados, conclui-se que o modelo teórico da Liderança Autêntica é altamente desejável e eficaz para o desenvolvimento do fator humano, levando resultados positivos nas organizações, incluindo instituições educacionais. Segundo a percepção dos professores, os três domínios da Liderança Autêntica foram considerados importantes para os cursos de Enfermagem nas universidades federais. Portanto, a adoção deste modelo teórico é vista como importante para o contexto educacional.

Este estudo apresentou algumas limitações, como o uso exclusivo de pesquisa *online* e amostragem por conveniência. Devido à heterogeneidade da amostra, os resultados não podem ser generalizados para professores que trabalham em universidades públicas federais de outras regiões do Brasil. Portanto, é necessário realizar pesquisas nacionais com amostras mais abrangentes para validar os resultados encontrados.

## Conclusão

Os resultados do estudo indicaram associação significativa positiva, sendo que a satisfação no trabalho dos professores nos cursos de graduação em enfermagem é influenciada pela prática da liderança autêntica por parte dos coordenadores, com destaque para as três variáreis: Relacional e Moral, Processamento Equilibrado de Informações e Autoconsciência. Isso confirma a hipótese levantada na pesquisa e sugere que comportamentos autênticos de liderança podem influenciar positivamente o ambiente de trabalho docente. Essa compreensão pode levar a uma melhoria na gestão, por meio de decisões mais assertivas no ambiente educacional.

O estudo realizado destaca a importância da liderança autêntica como fator significativo na satisfação no trabalho dos professores de graduação em enfermagem nas universidades públicas federais do Centro-Oeste brasileiro. Embora a associação seja modesta, a liderança autêntica, mesmo sendo um modelo teórico relativamente recente, possui uma representatividade significativa.

O domínio Relacional e Moral demonstrou relação significativa com a satisfação no trabalho dos professores, o que reforça a importância desses fatores para o constructo. No entanto, é importante lembrar que há outros fatores que influenciam a satisfação no trabalho e que precisam ser investigados em estudos futuros. A liderança autêntica estabelece uma base sólida para a adoção de modelos de liderança contemporâneos e inovadores no ambiente educacional, dessa forma, a orientação e integração contínuas são fundamentais para garantir um ambiente de trabalho positivo e satisfatório.

Os resultados obtidos confirmam a relevância da liderança autêntica no âmbito da educação, especialmente no contexto da educação em enfermagem, e abrem novas oportunidades para futuras pesquisas sobre modelos de gestão e liderança na área educacional. Além disso, esses achados proporcionam orientações valiosas para coordenadores, gestores e líderes educacionais, permitindo que baseiem suas práticas de gestão e liderança nos princípios da liderança autêntica. Isso, por sua vez, contribui para a promoção de relações de trabalho mais positivas e o aumento dos níveis de satisfação no ambiente de trabalho.
